# Prediction of dengue outbreaks in Mexico based on entomological, meteorological and demographic data

**DOI:** 10.1371/journal.pone.0196047

**Published:** 2018-08-06

**Authors:** Gilberto Sánchez-González, Renaud Condé, Raúl Noguez Moreno, P. C. López Vázquez

**Affiliations:** 1 Centro de Investigación Sobre Enfermedades Infecciosas, Instituto Nacional de Salud Pública, Cuernavaca, Morelos, México; 2 Departamento de Ciencias Naturales y Exactas, Universidad de Guadalajara, Ameca, Jalisco, México; Institut Pasteur, FRANCE

## Abstract

Dengue virus has shown a complex pattern of transmission across Latin America over the last two decades. In an attempt to explain the permanence of the disease in regions subjected to drought seasons lasting over six months, various hypotheses have been proposed. These include transovarial transmission, forest reservoirs and asymptomatic human virus carriers. Dengue virus is endemic in Mexico, a country in which half of the population is seropositive. Seropositivity is a risk factor for Dengue Hemorrhagic Fever upon a second encounter with the dengue virus. Since Dengue Hemorrhagic Fever can cause death, it is important to develop epidemiological mathematical tools that enable policy makers to predict regions potentially at risk for a dengue epidemic. We formulated a mathematical model of dengue transmission, considering both human behavior and environmental conditions pertinent to the transmission of the disease. When data on past human population density, temperature and rainfall were entered into this model, it provided an accurate picture of the actual spread of dengue over recent years in four states (representing two climactic conditions) in Mexico.

## Introduction

The infection rate of the dengue virus has shown a steady annual increase in Mexico, having potentially overcome acquired immune resistance [1]. Attempts have been made to explain epidemiological data with mathematical models of dengue infection. However, the introduction of climactic data has not led to reliable results. In Mexico, the great geographic variation of the inhabited regions makes the inclusion of this information an enormous challenge. Mexican geography ranges from seacoasts to areas 3 000 meters above sea level, and from desert to tropical climates.

The climactic diversity in Mexico provides fertile ground for the mathematical modelling of the transmission of dengue virus by *Aedes* mosquitoes. Real epidemiological data can be contrasted with the outcomes obtained from virus outbreak modelling. Mathematical models of dengue virus have been used to determine the impact of human intervention (in the environment and with therapy) on dengue infection pathology. The results of these models demonstrate that programs applied at the onset of symptoms, such as mosquito eradication techniques, have only marginally affected the rate of vector infection and virus transmission [2].

In an effort to model the risks factors of dengue transmission, Hopp and Foley simulated the mosquito life cycle and density in function of temperature and humidity. They suggested that mosquito density is the most important factor in dengue transmission [3]. Additionally, modeling efforts have considered the influence of geographic variation on dengue transmission [4], the expected effects of vector control policies [5], the climatological relationship with disease outbreaks [6], statistical analysis and projection of dengue transmission [7], and the assessment of individual infection risk as a function of location [8].

Dengue transmission models based on differential equations take certain aspects of disease transmission dynamics into account. Estevan et al. developed a model by employing the theory of competitive systems, compound matrices and the center manifold theorem, estimating that global cases of dengue would reach asymptotic stability [9]. Since the latter approach uses data from past conditions, it cannot adapt to the day-to-day meteorological information or updated epidemiologic data, making it impossible to determine changes in virus biology. The continuous state of climactic changes and human population dynamics have made the prediction of that model elusive [10].

Despite the complex geographic and climactic features of Mexico, analysis of the spread of the dengue virus has been linked only to mosquito density, human population density, and mosquito and human transmission rates in both directions (infected mosquitoes to humans and infected humans to mosquitoes). We herein present a mathematical model that considers most of the biological factors having an influence on dengue virus infection dynamics, both in mosquitoes and humans.

## Materials and methods

### The model

A non-linear time-delayed differential equation system was developed to model the seasonal life cycle of mosquitoes and their interaction with humans. This model differs from previous approaches because it contemplates the survival of mosquito eggs through the drought season and the existence of a vertical transovarial infection from infected female mosquitoes to their eggs. The model weighs the effective bite rates of mosquitoes and the non-constant proportions of susceptible humans and infected mosquitoes. It also includes parameters that are dependent on temperature (*T*), rainfall (*P*) and time (*t*). These parameters have been concisely selected and calculated in accordance with reported mosquito behavior and biology, such as the developmental timing of the aquatic stages, the life expectancy of the adult mosquito, the amount of eggs that can be laid, and the extrinsic incubation period (see S1 Supporting Information for details).

**Fig 1:**
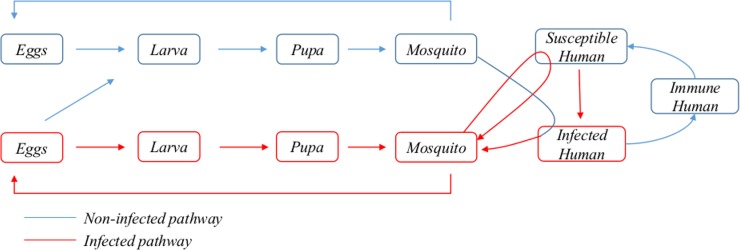
Scheme of the relationship between the biological actors in the model.

The model comprises twelve different populations that evolve over time. The insect population refers to female *Aedes* mosquitoes. The population variables are as follows: *x*_*1*_ and *x*_*2*_ correspond to non-infected and infected eggs, respectively; *x*_*3*_ and *x*_*4*_ to non-infected and infected larva; *x*_*5*_ and *x*_*6*_ to non-infected and infected pupa; *x*_*7*_ and *x*_*8*_ to non-infected and infected mosquitoes; *x*_*9*_ and *x*_*10*_ to infected and immune humans; and *x*_*11*_ and *x*_*12*_ to non-infected and infected resting eggs. The latter factor is crucial for the resurgence of disease in regions with extremely dry summers. Infected human populations comprise symptomatic and asymptomatic individuals, both of which are infective for mosquitoes. Once a person overcomes an infection, he or she is assumed to remain immune, and therefore cannot be a carrier (human vector), for a certain period of time (presently, this period is set at six years). The model considers that a recently infected mosquito can only be infective upon the passage of a period of time τ, which corresponds to the extrinsic incubation period (EIP). Likewise, recently infected humans can only be infective for the mosquitoes after a certain period of time λ. A schematic representation of the model is depicted in Fig 1.

Distinct dengue virus serotypes have different EIP and infectivity [11]. The biological data from the serotype one virus (DENV-1) was used herein because it is the most prevalent in Mexico [12]. In our model, the total human population (*H*) remains constant over time. The system of ordinary differential equations is as follows.

System of differential equations

$$\frac{dx_{1}(t)}{dt}=k_{1}[x_{7}(t)+(1-m)x_{8}(t)]+k_{18}x_{11}(t)-(k_{2}+k_{17}+k_{3})x_{1}(t)$$ (1)

$$\frac{dx_{2}(t)}{dt}=m\;k_{1}x_{8}(t)+k_{18}x_{12}(t)-(k_{2}+k_{17}+k_{3})x_{2}(t)$$ (2)

$$\frac{dx_{3}(t)}{dt}=k_{2}x_{1}(t)-(k_{4}+k_{5}+k_{6}x_{3}(t))x_{3}(t)$$ (3)

$$\frac{dx_{4}(t)}{dt}=k_{2}x_{2}(t)-(k_{4}+k_{5}+k_{6}x_{4}(t))x_{4}(t)$$ (4)

$$\frac{dx_{5}(t)}{dt}=k_{5}x_{3}(t)-(k_{7}+k_{8})x_{5}(t)$$ (5)

$$\frac{dx_{6}(t)}{dt}=k_{5}x_{4}(t)-(k_{7}+k_{8})x_{6}(t)$$ (6)

$$\frac{dx_{7}(t)}{dt}=k_{7}x_{5}(t)-(k_{9}x_{9}(t)+k_{10})x_{7}(t)$$ (7)

$$\frac{dx_{8}(t)}{dt}=k_{7}x_{6}(t-\lambda )+k_{9}x_{9}(t-\lambda )x_{7}(t-\lambda )-k_{11}x_{8}(t)$$ (8)

$$\frac{dx_{9}(t)}{dt}=k_{12}\frac{l[H-(x_{9}(t)+x_{10}(t))]x_{8}(t)}{s(x_{9}(t)+x_{10}(t))+l[H-(x_{9}(t)+x_{10}(t))]}-(k_{13}+k_{14})x_{9}(t)$$ (9)

$$\frac{dx_{10}(t)}{dt}=k_{13}x_{9}(t)-(k_{15}+k_{16})x_{10}(t)$$ (10)

$$\frac{dx_{11}(t)}{dt}=k_{17}x_{1}(t)-(k_{18}+k_{19})x_{11}(t)$$ (11)

$$\frac{dx_{12}(t)}{dt}=k_{17}x_{2}(t)-(k_{18}+k_{19})x_{12}(t)$$ (12)

With $l=\frac{H-(x_{9}(t)+x_{10}(t))}{H}$ and ***s* = 1 − *l***. In the model, the parameters *l* and *s* control for the percentage of mosquito bites in susceptible humans, representing the vector-bias effect. More specifically, *l* and *s* denote the probability that an arriving mosquito will bite an infected and immune person, respectively. The *x* variables and *k* parameters are described in Table 1.

**Table 1:** Values and description of the variables and parameters of the model. *T*_*in*_ and *T*_*out*_ designate the indoor and outdoor temperature, respectively. *P* is the rainfall and *θ(X)* is the Heaviside step function whose value is zero for a negative argument and one for a positive argument (see S1 Supporting Information for details of the calculation of parameters).

Variable	Interpretation		
*x*_*1*_	Non-infected eggs		
*x*_*2*_	Infected eggs		
*x*_*3*_	Non-infected larvae		
*x*_*4*_	Infected larvae		
*x*_*5*_	Non-infected pupae		
*x*_*6*_	Infected pupae		
*x*_*7*_	Non-infected mosquitoes		
*x*_*8*_	Infected mosquitoes		
*x*_*9*_	Infected humans		
*x*_*10*_	Immune humans		
*x*_*11*_	Resting non-infected eggs		
*x*_*12*_	Resting infected eggs		
Initial conditions			
*x*_*1*_*(0) = 0*, *x*_*2*_*(0) = 0*, *x*_*3*_*(0) = 0*, *x*_*4*_*(0) = 0*, *x*_*5*_*(0) = 0*, *x*_*6*_*(0) = 0*, *x*_*7*_*(0) = 0*.*99*, *x*_*8*_*(0) = 0*.*01*, *x*_*9*_*(0) = 0*, *x*_*10*_*(0) = 0*, *x*_*11*_*(0) = 0*, *x*_*12*_*(0) = 0*			
Parameter	Interpretation	Value	Reference
*H*	Human density	3.7	[13]
*k*_*1*_*(T(t))*	Mosquito oviposition rate	k_12_ (-71.06 + 7.59 T_out_− 0.14 T_out_^2^)/2	[14], [15], [16], [17]
*k*_*2*_*(T(t))*	Rate of progression to the larval stage	(37.06–2.08 T_out_− 0.03 T_out_^2^)^-1^	[17])
*k*_*3*_*(T(t))*	Mortality rate of eggs (during the rainy season)	0.38 k_2_	[17]
*k*_*4*_*(T(t))*	Rate of progression to the pupal stage	(55.49–2.86 T_out_− 0.04 T_out_^2^)^-1^	[17]
*k*_*5*_*(T(t)*,*P(t))*	Mortality rate of larvae	0.25 δ k_4_	[17]
*k*_*6*_	Density-dependent mortality rate of larvae	0.05	[18]
*k*_*7*_*(T(t)*, *P(t))*	Rate of progression to mosquito stage	(18.78–1.00 T_out_− 0.01 T_out_^2^)^-1^	[17]
*k*_*8*_*(T(t))*	Mortality rate of pupae	0.09 k_7_	[17]
*k*_*9*_	Infectious meal rate from humans to mosquitoes	0.3	[19], [20]
*k*_*10*_*(T(t))*	Mortality rate of healthy mosquitoes	(-90.76–9.54 T_out_− 0.18 T_out_^2^)^-1^	[17]
*k*_*11*_*(T(t))*	Mortality rate of infected mosquitoes	1.56 k_10_	[21]
*k*_*12*_*(T(t))*	Infectious bite rate from mosquitoes to humans	0.2 (1 –k_11_ τ) ϴ(1 –k_11_ τ)	[17], [15], [22]
*k*_*13*_	Infected human death rate	0.99 k_15_	[23], [24]
*k*_*14*_	Immunity acquisition rate	0.14	[25]
*k*_*15*_	Human death rate	6.5 10^−7^	[15]
*k*_*16*_	Immunity loss rate	4.5 10^−4^	[26]
*k*_*17*_*(P(t))*	Mosquito emergence deactivation by drought	1—k_18_	Supposed
*k*_*18*_*(P(t))*	Mosquito emergence activation by rain	ϴ(P– 1)	Supposed
*k*_*19*_	Mortality rate of eggs during drought	0.018	[27]
*τ*	EIP	600 (0.3/2π)^1/2^ Exp(-0.3 (T_in_− 5.9)^2^ /T_in_)	[11]
*λ*	Dengue incubation period in humans	3	[20]
*δ(P(t))*	Rainfall-dependent ponderations	1 –(0.1389–0.0136 P)	[28]

### Different outcomes and scenarios of the model

The equations of the model are solved by using Wolfram Mathematica 8. The density of humans and mosquitoes as well as the incidence of infected humans and mosquitoes are expressed per household unit. The calculations were made for two case scenarios as a function of temperature rainfall. The first hypothetical scenario was constructed by defining the rainfall input (rainy season) as a sinusoidal function with a constant period and intensity (amplitude), while the temperature remained fixed. In the second hypothetical scenario, we considered weekly temperature and rainfall data from eight consecutive years, obtained from meteorological stations in four cities in Mexico.

### Differentiation between indoor and outdoor temperatures

The distinct indoor and outdoor temperature is relevant herein because adult mosquito life is mainly spent indoors and the aquatic stages develop outdoors. For our simulations, outdoor temperatures were taken from the respective meteorological station and the indoor temperature was correlated with this. For the latter, there was a region-dependent minimum temperature *T*_*min*_. The maximum indoor temperature was also correlated with the outdoor temperature, but with a pull-down factor that reduces peak values, as observed in the real world [29]. The indoor temperature *T*_*in*_ is thus defined as:

$$T_{in}=\left\{ \begin{array}{c} T_{min},\;T_{out}<T_{min}\\ T_{out}+(1-1.3\;e^{-\frac{T_{out}-T_{min}}{2}}),\;T_{out}\geq T_{min} \end{array}\right.$$ (13)

### Epidemic prediction capacity of the present model after integrating meteorological and demographic data

The predictive power of the model was evaluated by entering the weekly temperature and rainfall data over the last eight years from four Mexican cities (in four different states), and comparing the projected dengue transmission with the number of confirmed dengue infections per year reported by the corresponding state health institutions. To establish a stable set of parameters calculated from the model, real data from 2008–2012 were used. The estimated values for 2013–2016 were then compared to the epidemiological records. To test the behavior of the model, we selected four dengue-endemic cities from two regions with distinct climates. The weather in the cities of Guadalajara and Colima is hot and dry, while that in Tuxtla Gutierrez and Campeche is hot and humid.

The real incidence of infection is influenced by several natural and socio-economic factors that vary within and between regions and over time. Regarding entomological factors, the chemical control interventions (the most common for dengue control in Mexico) distort the base line of epidemiological data for mosquito density. Although the impact of these interventions is hard to determine, we saw little difference between chemically-treated areas and those with no chemical intervention [30]. This observation justifies performing a direct comparison between the calculated values of the model and the epidemiological data.

The approach of the study is briefly explained. For any given time *t*, the observed number of dengue patients was assumed to be correlated with both the local healthcare coverage (taking the number of doctors per habitant as a reliable indicator) and the proportion of symptomatic cases, the latter estimated for the most prevalent serotype, DENV-1. Thereby, the *observed incidence* was computed as (model-projected incidence)*(proportion of symptomatic cases)*(healthcare coverage)*(number of houses). The density unit used here is mosquitoes and persons per household unit. Hence, to obtain the total number of dengue cases, the corresponding density was multiplied by the number of houses in the entity.

The dynamics governing the infection and propagation of the dengue virus as well as the severity of the epidemic are complex and specific to the particular human and environmental factors in any given locality. A homogeneous surface unit was adopted for the model, in which precipitation, temperature, and mosquito and human density were taken into account to simulate a dynamic population. In this fashion, distinct scenarios were predicted.

### Ethics statement

The present research was completed in accordance with the INSP (National Institute of Public Health in Mexico) ethical guidelines, and no experimental work was performed. The authors have no conflict of interest in the materials or procedures utilized in the study.

## Results

### General properties of the model

**Fig 2:**
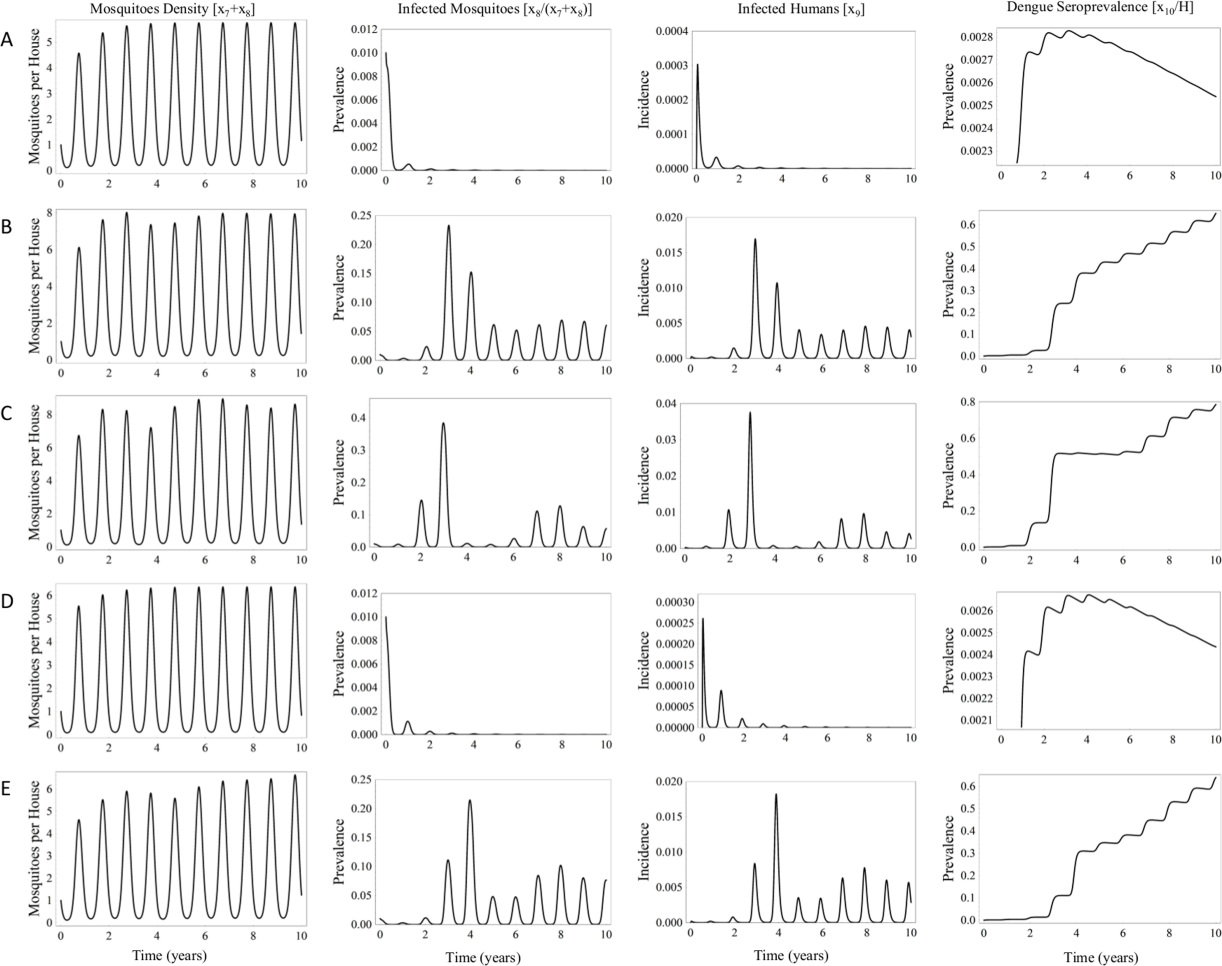
Outcomes of the model for various temperatures: (A) 26°C, (B) 28°C, (C) 30°C, (D) 32°C, and (E) a temperature transition from 26–27°C. Dengue virus transovarial transmission is set at zero.

Considering that transovarial transmission of the dengue virus is still a contentious issue, we initially set this factor to zero. The calculated values of the dengue transmission model are shown for different temperatures (Fig 2). The average annual temperature chosen for any given simulation ranged from 26–32°C, which is the reported variation among the four virus-endemic cities herein studied. Temperature is a relevant factor for dengue transmission.

First, calculations were made for a temperature of 26°C, finding the mosquito density, prevalence of dengue-infected mosquitoes, prevalence of dengue-infected humans, and prevalence of seropositivity in the population (Fig 2A). The model predicts a maximum average number of mosquitoes per house of five and a minimum value less than one. In the scenario of mosquito density close to one, the model estimates that dengue incidence decreases to a steady state close to eradication, although it does not disappear within a ten-year period.

The same parameters were calculated for a temperature of 28°C (Fig 2B). The mosquito density increases slightly to an average maximum of seven mosquitoes per house. During a dengue outbreak, the number of infected mosquito peaks at 60% during the third year. The model projects the prevalence of dengue in humans of approximately 6%. In addition, the average estimated prevalence of human dengue seropositivity reaches 40% after four years.

With the temperature set at 30°C, the results are similar to those found for 28°C (Fig 2C and Fig 2B), except that the peaks of dengue infection last less time. The pattern of epidemic dynamics is interesting, with seropositivity reaching a transitory plateau of approximately 50% at 3 years of the epidemic, and then rising again after six years. This is probably due to the loss of immunity, on the average, of the humans who are infected during the very first waves of projected infection. Hence, despite the fact that the mosquito density remains roughly the same between 28 and 30°C, the pattern of the epidemic is different. The distinct pattern can be explained by the temperature dependence of the equations determining mosquito development, survival and life cycle. The temperature affects mosquito oviposition time, post blood alimentation, and the time elapsed before a mosquito is capable of infecting the host (i.e., the time needed for the virus to reach the salivary glands). These factors are optimum for the mosquito vector when the simulation is executed with the temperature set between 28°C and 30°C. Under such conditions, the EIP is the shortest and the mosquito life expectancy the longest.

For a temperature set at 32°C (Fig 2D), mosquito larvae undergo a shorter development time in the aquatic state and a lower mortality. The life expectancy of the mosquito terrestrial stage decreases. The sum of these opposite effects results in a very similar mosquito density when comparing 32°C with 28–30°C. Nevertheless, the shorter mosquito life expectancy at the higher temperature (32 ºC or more) influences the dynamics of virus transmission, due to the reduced probability that an adult mosquito will live long enough to be able to infect a human host.

The effects of a 1°C temperature increase (from 26 to 27°C) is projected for the dengue infection over ten years (Fig 2E), predicting a reactivation of the epidemic. This increment in temperature creates more suitable conditions for dengue transmission because mosquito eggs in resting state can survive for longer periods of time. The postulated existence of a pool of transovarially-infected mosquitoes could potentially lead to an outbreak in regions where dengue was supposedly eradicated. Finally, another important feature of the calculated values of the model shown in Fig 1 is the presence of peaks and valleys in the incidence of infection, indicating rich internal dynamics. This reveals that meteorological variables are the main driving force of the model, resulting in periodic patterns of the initial assessment.

**Fig 3:**
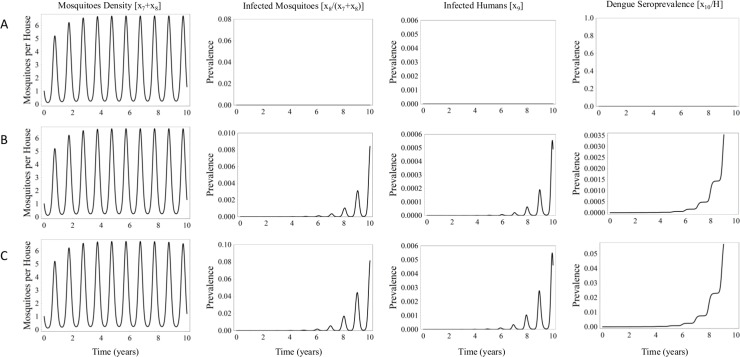
Simulations with the model by using three transovarial transmission values: (A) the reference scenario, with no vertical transmission, (B) 1/1 000 eggs infected and (C) 1/100 eggs infected. The temperature was set at 28°C.

As an exploratory exercise, we calculated the outcome of the model using different rates of dengue transovarial transmission (Fig 3). The initial conditions computed by the model start with a mosquito density of zero, an egg density of 0.01 eggs per square meter, and all humans considered susceptible to dengue infection. With the temperature fixed at 28°C, the direct effect of zero transovarial dengue virus transmission was analyzed (Fig 3A), finding the expected value of mosquito density at its normal level and no epidemic. Another scenario was contemplated in which 1/1 000 eggs are originally infected and 1/1 000 eggs laid by infected females carry the virus (Fig 3B). The mosquito density reaches its normal level, but there is a slow and gradual epidemic resurgence, reaching a proportion of seroprevalence in humans of 35/10 000 after ten years. Consideration was also given to a scenario in which 1/100 eggs are originally infected, and 1/100 eggs laid by infected females carry the virus (Fig 3C). The mosquito density again reaches its normal level, but the dynamics of the epidemic leads to the seroprevalence in humans of 5/100 at the tenth year. These results suggest that dengue vertical transmission has the potential of producing a notable resurgence in an epidemic.

### Mosquito density

**Fig 4:**
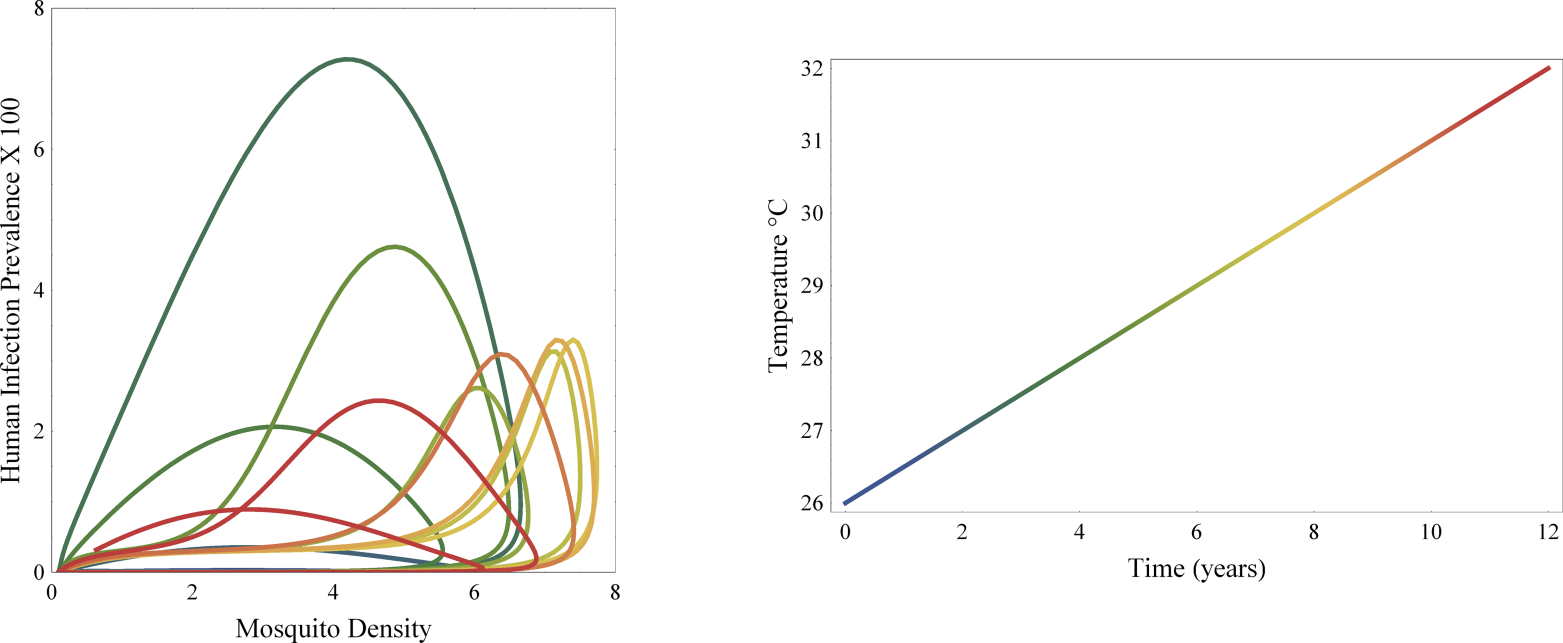
Phase diagram of the variations in mosquito density and the incidence of human infection as a function of temperature. Dengue virus transovarial transmission is set to zero.

A phase diagram of human infection prevalence versus mosquito density was constructed (Fig 4). The most important predicted pattern of human dengue infection in the epidemic is that its intensity is not strongly dependent on mosquito density, unless such density borders on eradication. This unexpected outcome can explain the inadequacy of dengue eradication programs based on truncated insecticide/larvicide campaigns.

The relation between human infection and mosquito density changes as the temperature rises from 26 to 32°C. With an increase from 26 to 28°C, the model produces concentric orbits aligned with a mosquito density of 4 female mosquitoes per house. The most suitable conditions for dengue virus transmission occurs at 28°C, finding more infections per mosquito. Between 30 and 31°C, the model projects a greater density of mosquitoes but with less effect on transmission. Again, the model shows that mosquito density is not strongly correlated with the incidence of disease.

In the present simulation, mosquito density has less influence on transmission than in other models. The temperature dependence of a dengue epidemic could account for the apparent contradiction between the observed rise in dengue cases in countries where the density of mosquitoes has been reduced by fumigation and vector control measures [30]. The epidemic expansion may have been due to the fact that simultaneous with diminished mosquito density was a change from slightly to highly permissive local temperature.

### Model simulation of past epidemics using meteorological data

**Fig 5:**
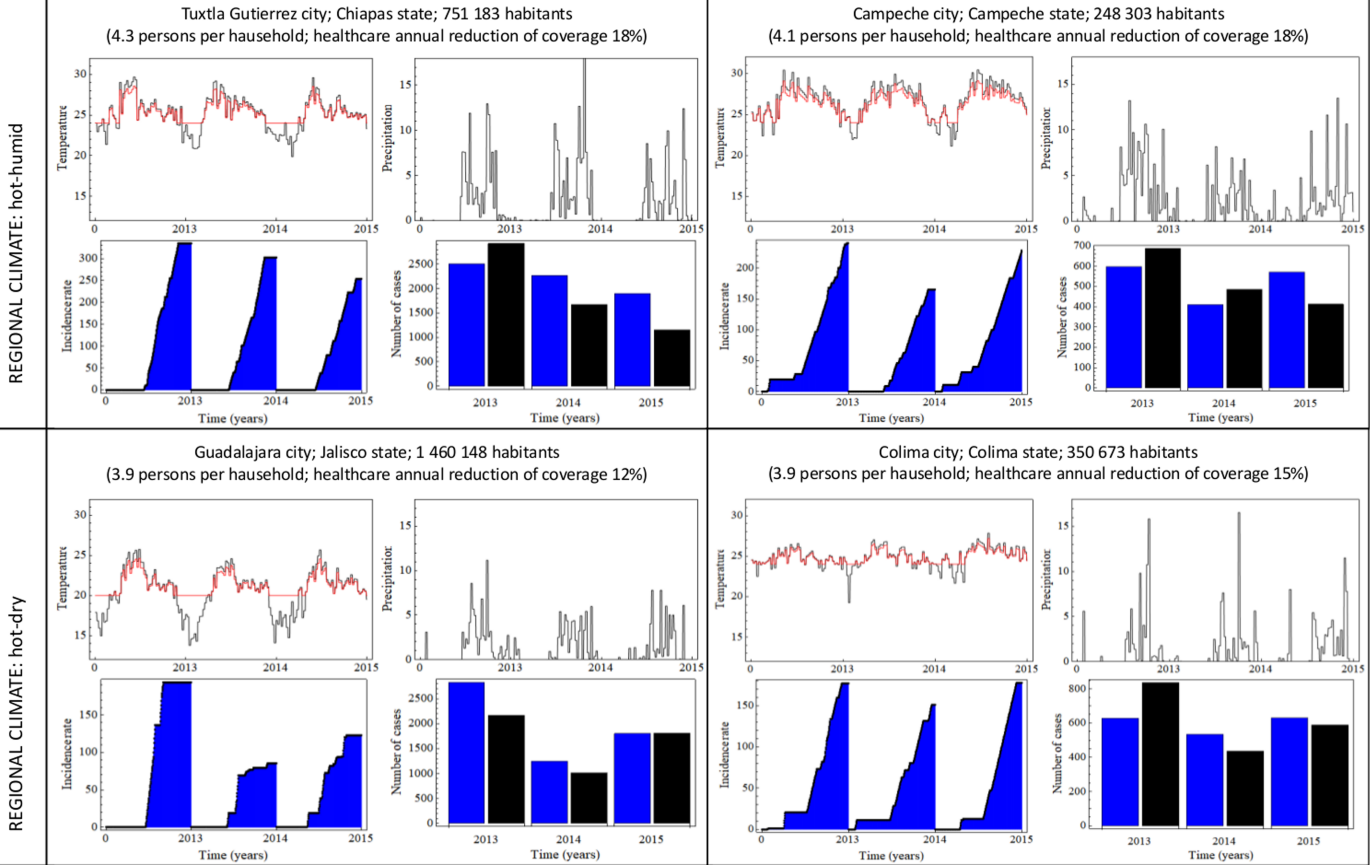
Predictions of dengue infections based on the current model by using past meteorological and demographic data. For the histogram, the blue bars portray the projections made by the model and the black bars the observed epidemiological data. In the temperature graph, the black lines correspond to the outdoor temperatures and the red lines to the indoor temperatures. Historical data on epidemics were gathered from the Epidemiological History Bulletin available at https://www.gob.mx/salud/acciones-y-programas/historico-boletin-epidemiologico. Demographic data were taken from the National Household Survey 2015 (available at: http://www.inegi.org.mx/saladeprensa/boletines/2016/especiales/especiales2016_06_05.pdf.) and from the National Statistical and Geographic Information System (available at http://cuentame.inegi.org.mx/monografias/default.aspx?tema=me). Healthcare coverage information was obtained from the CONEVAL (available at evaluation of social policy http://www.coneval.org.mx/Evaluacion/Paginas/Indicadores_de_acceso_y_uso_efectivo_de_los_servicios_de_salud_de_afiliados_al-Seguro_Popular.aspx.). Meteorological data (temperature and rainfall) were downloaded from the corresponding meteorological stations by the Mathematica 8 “WeatherData” centers. The proportion of symptomatic cases for DENV-1 was taken as 1/11 [31]. Dengue virus transovarial transmission was set to zero for these calculations. In the graph, the temperature unit is Celsius degrees and the rainfall is millimeters of rain.

To evaluate the precision of the model for accurately simulating dengue transmission over time, we ran the model with the information on rainfall and temperature reported in the four cities under study during seven years. As mentioned in the previous section, the total annual dengue infections estimated by the model were further adjusted to take into account 1) the proportion of symptomatic cases, 2) healthcare coverage and 3) the number of houses in each city. When the projected prevalence of dengue was compared to the actual recorded prevalence (according to the Mexican Health Department data) during the latter three years, the number of dengue infections was closely predicted. Furthermore, the year to year patterns of infection (increases or decreases) were also accurately predicted. Hence, the results confirm the reliability of the present model for projecting dengue infections in the two different regional climates involved in the study (Fig 5).

## Discussion

The dengue epidemic is one of the most mathematically modeled among infectious diseases. We herein compare the current epidemiological mathematical model to others based on differential equations. With the introduction of complexity, previous models tend to become chaotic, unstable and difficult to control, as evidenced by three reported models with a great number of mathematical relations and equations [32, 33, 34]. Such difficulties explain why many authors have tried to achieve stability in their model by reducing the “noise” in equations (i.e., omitting meteorological data). Contrarily, historical meteorological data and initial epidemiologic conditions are presently included, thus more closely simulating real-time dengue epidemic conditions. This likely explains the improved capacity of our model for predicting outbreak probability. The results reported herein demonstrate that feeding the model with accurate day-to-day data could provide a valuable tool for public health decision makers.

Nowadays, the importance of temperature and rainfall for mosquito development and population maintenance is well documented. Several authors have added these parameters to their dengue transmission models, either setting them at fixed values or permitting slight variations over time [35]. Transovarial transmission, which is now gaining recognition, has been included in some mathematical models [36, 37]. Despite contemplating these elements, previous models have not successfully integrated them into a coherent equation system. The current model represents a relevant validated synthesis of many previous dengue modeling attempts.

Special emphasis was placed on the temperature and rainfall dependence of the following mosquito-related factors: the oviposition rate, aquatic development, mosquito bite rate, extrinsic infection period, and mortality rate. Three aspects left out by other authors were introduced in the present model: the existence of a resting state for mosquito eggs during the drought season, the use of two distinct temperatures (indoor for the behavior of adult mosquitoes and outdoors to model the aquatic cycle of development), and the insertion of real meteorological data.

## Conclusions

We elaborated and verified an epidemiological mathematical model of dengue transmission that takes environmental variations into account. This model could be utilized to establish a dengue risk prediction map, potentially enabling governments to implement timely public health actions to diminish the impact of epidemics. Interestingly, a dengue outbreak was herein found to depend less on mosquito density than on environmental temperature.

The model includes the potential factor of vertical dengue transmission because it could possibly explain dengue outbreaks in regions where the virus had apparently been eradicated for several years. The impact of such dengue propagation is limited in this model unless the percentage of transmission reaches 1% at the optimal temperature of 28–30°C (see Fig 3), a percentage never before recorded experimentally. Nevertheless, there is always the possibility of a butterfly effect that would amplify the impact of a vertical transmission event.

The transmission of dengue over long distances should be attributed mainly to human displacement, meaning that humans represent the actual dengue vector. *Aedes aegypti* mosquitoes are broadly recognized as peri urban, moving about within a mere 120-meter radius over their lifetime, on the average [38]. Although transmission is indeed mediated by the presence of mosquitoes, dengue transmission is inevitably linked to human interurban movements.

The Mexican territory comprises vast areas, including rain forests in the southern states of Chiapas and Yucatan, where the climatic conditions allow for a year-long active mosquito life cycle. On the other hand, a large portion of the central region, such as the states of Mexico and Jalisco, have a six-month dry season. The continuous movement of people between the latter two states alone would outweigh the effect of transovarial dengue transmission on disease outbreaks. One clear example occurred in 2014, in which 160 000 individuals were displaced within the country (idmc, Global Overview 2014: People Internally displaced by conflict and violence, May 2014).

The outcomes of the model as a result of a rise in temperature are particularly striking. The optimal temperature for dengue transmission is 28–30°C. In recent years, there has been a transition to a more permissive temperature (25–27 ºC) in several regions of the world (27), triggering consequences for dengue transmission. The model predicts that even if the mosquito density diminishes with a higher temperature, transmission will still remain because of more optimum biological factors for the dengue virus. Hence, global warming will affect dengue transmission, as more regions will reach an optimum transmission temperature in the coming years.

## Supporting information

S1 Supporting Information (DOCX)
