# Supplementary material for: Prediction of dengue outbreaks in Mexico based on entomological, meteorological and demographic data
Source: PLoS One. 2018 Aug 6;13(8):e0196047. doi: 10.1371/journal.pone.0196047 (PMC6078291; doi:10.1371/journal.pone.0196047)
Supplement: S1 Supporting Information — (DOCX) [file pone.0196047.s001.docx]

The aim of this supplementary material is to describe extensively the Table 1 parameters calculations. We will only describe the parameters that are created utilizing several values obtained from different sources of the literature. Also, the equations related to transovarical transmission and the infection in the human are further discussed.

**Mosquitoes´ oviposition rate (k_1_ parameter)**

The oviposition rate is known to be a consequence of previous blood meals and the quantity of laid eggs is also affected by temperature. The number of eggs laid in function of temperature were adjusted by polynomial approximation of the data from Panigrahi et al. S1 [1] resulting in the equation$-71.06+7.59 T_{\mathrm{out}}-0.14 {T_{\mathrm{out}}}^{2}$. This equation is multiplied by the k_12_ parameter (the rate of meals which correlate with oviposition), which determines the oviposition occurrence rate. Since only female progeny are taken into account, the result is divided by 2. The resulting equation is $\frac{k_{12}}{2}\left( -71.06+7.59 T_{\mathrm{out}}-0.14 {T_{\mathrm{out}}}^{2} \right)$.

**Development rates in aquatic phase (k_2_, k_4_ and k_7_ parameters)**

The development rates from egg to larvae, pupae and mosquito stage as a function of temperature were adjusted by polynomial approximation from the data of Panigrahi et al. S1 [1]. The resulting equations are $k_{2}=\left( 37.06-2.08 T_{\mathrm{out}}+0.03{T_{\mathrm{out}}}^{2} \right)^{-1}, k_{4}=\left( 55.49-2.86 T_{\mathrm{out}}+0.04{T_{\mathrm{out}}}^{2} \right)^{-1}, k_{7}=\left( 18.786-1.00 T_{\mathrm{out}}+0.01{T_{\mathrm{out}}}^{2} \right)^{-1}$.

**Mortality rates in aquatic phase (k_3_, k_5_ and k_8_ parameters)**

The mortalities rates of eggs, larvae and pupae stage as a function of temperature were also calculated from S1 [1] as the proportion of the population which accomplish each development phase, divided by the average duration of the corresponding aquatic phase. The resulting equations are $k_{3}=0.38 k_{2}, k_{5}=0.25 \delta k_{4}, k_{8}=0.09 k_{7}$, were $\delta$ is a rainfall ponderation to be described ahead.

**Infectious rate from humans to mosquitoes (k_9_ parameter)**

This parameter is constructed from the probability that an infected human got bitten by a healthy mosquito multiplied by the host peak viremia duration. From Harrington el al. S1 [2] we know that the probability of being bitten is 0.67. On the other hand, from Guzman et al. S1 [3] we know that the peak of the viremia last for over 4 days of the 7 days of the duration of the infection S1 [4]. Given these conditions, we set the probability of infection for the mosquito equal to 0.8, since typical viremias peaks are of 8 log_10_ copies per millilitre in average S1 [5]. The final value for k_9_ is k_9_ = 0.67*(4/7)*0.8 = 0.3.

**Mortality rates of mosquitoes (k_10_, k_11_ parameters)**

The mortalities rates of healthy mosquitoes were obtained from S1 [6] using polynomial approximation of his data. The resulting equation was $k_{10}=\left( -90.66+9.54 T_{\mathrm{in}}-0.18 {T_{\mathrm{in}}}^{2} \right)^{-1}$. The mortality rates of infected mosquitoes was obtained by odds ratios from Maciel-de-Freitas et al. S1 [7]. The resulting relation was k_11_ = 1.56 k_10_.

**Infectious bites rate from mosquitoes to humans (k_12_ parameter)**

This parameter is constructed multiplying the probability of mosquito to be infected by the probability of a mosquito to bite a person multiplied by the probability of the bite to be infective. From S1 [8], we know that the probability that an infected mosquito is going to be searching for a host is 0.6. The probability that the mosquito is infective is determined by the IEP and its life expectancy. The effective time of dengue transmission for the mosquito is 1/k_11_-τ, and to obtain the probability, it must be multiplied by k_11_. On the other hand, the distribution of the number of different types of bloods observed in mosquitoes coexisting with humans was obtained from Harrington et al. S1 [2]. From this publication, we determined that the probability of being bitten is 0.33 and the average number of bites to different persons from the same mosquito is set at 2.1 (see “The mosquito Aedes aegypti feeding behaviour and timing” section). Finally, as we already know, the probability of virus transmission depends of the viral load presented by the mosquito, and as long as this levels are unknown we set the probability of transmission conditioned to be out of the EIP period, to 0.5. The final equation is k_12_ = 0.6*0.33*2.1*(1/k_11_-τ) k_11_*0.5 = $0.2 (1-k_{11}\tau)\theta(1-k_{11}\tau)$_._ The theta unit-step function prevent negative arguments if the IEP is longer that the life expectancy of the mosquito.

**The mosquito Aedes aegypti feeding behaviour and timing**

Commonly, the female *Aedes aegypti* is capable of blood feeding 24 hours post emergence S1 [9]. After feeding, the female mosquito enters a 60 hour digestion cycle that leave the insect in a quiescent state S1 [10]. The infection affects the mosquito feeding behaviour, increasing by 5% the probing time. Nevertheless, this extension of the probing time has a marginal effect on transmission S1 [11]. Mosquitoes collected from the wild have been reported to present a maximum of 12 different human blood in their stomach, having therefore had a maximum of 12 theoretical feeding event for one female. We considered the number of feeding events of the result table from Harrington et al. S1 [2] to calculate the average number of mosquito blood meals from different persons. The value of this constant was 2.5.

**Virus infection in the mosquito (τ parameter)**

The virus extrinsic incubation period is also temperature dependent and, at temperature below 20ºC, the EIP equation is $600\sqrt{\frac{0.3}{2\pi}}Exp(-0.3\frac{{(T_{in}-5.9)}^{2}}{T_{in}})$ is nearly equal to the mosquito lifespan S1 [11]. The virus may impact the feeding behaviour of the mosquito through antennae sensory protein alteration, leading to an increase number of probing before successful blood taking, hence potentially altering the transmission pattern of infected mosquitoes S1 [12]. The number of different host DNA found in a *Aedes aegypti* female body was tested by PCR reaction. Researchers encountered an exemplary with DNA traces corresponding to 11 human genomes. The average mosquitoes would have 3 bloods feeding during their lifetime S1 [13].

**Rainfall-dependent ponderation (δ parameter)**

The rainfall intensity affects mosquito population, hence the Dengue epidemic propagation. There are optimum conditions for the development and survival of the progeny of the mosquitoes. For instance, lower rainfall affects larvae development and survival and heavy rainfall produces high mortalities by flash floods dragging effects. In this context, we introduced a rainfall coefficient to adjust the mosquito density, using a polynomial equation based on the data published by Ehelepola S1 [14]. The ponderation is $\delta=1-(0.1389-0.0136 P)$, were P is the rainfall quantify in millimetres.

**Transovarical transmission of dengue virus**

The eventuality of virus transovarical transmission has been considered to be an important aspect of the epidemiology of mosquito-borne viruses. This phenomenon would explain the dengue virus resurgence after dry seasons (or winter) or other adverse environmental periods where adult arthropod hosts are absent. During these interludes, the virus permanency would be mediated by the eggs containing the virus, passing it from one insect generation to the other S1 [2]. Since *Aedes* mosquitoes do not migrate great distances from their breeding site (their average displacement radius is 120 m), the cartography of the diversity and distribution of circulating DENV serotypes present in mosquitoes constitutes an essential information to evaluate the risk of transmission of a specific DENV serotype S1 [1]. Data about Dengue virus vertical transference are detailed in about 20 research publications. Reported mosquito eggs infection rates range from 2.3% to 0.01%. Although the virus genome or NS1 protein have been found in eggs or larvae, it has not been demonstrated that the virus forms infectious particles. There are no convincing evidences that active virus is found in the salivary glands of the adult mosquito coming from infected eggs; neither that this virus would be capable of infecting the vertebrate host. Therefore, in this model; we will consider that the transovarical virus transfer ratio is 0.01%. Nevertheless, a transmission mechanism in which the DENV would remain dormant during adverse periods for the insect vector *Aedes aegypti* would explain the generation of new epidemic outbreaks in isolated population after long relapse periods.

**Human infection**

In human, the infection by dengue virus present an incubation period of three to eight days, before the onset of the symptoms. The febrile period usually resolves by the fifth day of symptomatic dengue, though the febrile period ranges from one to eight days, depending on the infected human health status and Dengue serotype S1 [6]. The human blood Dengue virus viremia peaks at 10^8^ to 10^9^ virus per ml for 4 days and show viremia above 10^6^ virus per millilitre of blood during four days post symptomatic infection S1 [15] S1 [16]. Duong and col. determined that the asymptomatic/pre-symptomatic human dengue stage of infection are up to 100 times more infectious to the mosquito than symptomatic human virus carrier S1 [17]. The human blood virus title that allow Dengue transmission to the mosquito has been experimentally determined and oscillate between 10^6^ to 10^8^ pfu/ml in order to have 50% of the fed mosquitoes infected S1 [18]. Another publication do set the blood viremia lower limit for mosquito effective infection to 10^6^ pfu S1 [19].

**Code of the program**

The code is compatible with Wolfran´s Mathematica 8 or latter:

AnosSim=3;(*Period of time to be compared with data*)

AnosEstabilizar=4;(*Period of time to stabilize the solutions*)

loops=(AnosSim+AnosEstabilizar)*365;

(*Auxiliar variables*)

Sol1={};

Sol2={};

Sol3={};

Sol4={};

Sol5={};

Tempera={};

Pluvio={};

Temperatura={};

Lluvias={};

Temporal1={};

Temporal2={};

(*Demographic and epidemiological data*)

TamanoPob=248303; (*Total population*)

H=4.1;(*Persons per house*)

TR1=764;(*Observed cases year 1*)

TR2=540;(*Observed cases year 2*)

TR3=458;(*Observed cases year 3*)

DismCob=0.18;(*% annual reduction health coverage*)

Registro=0.12;(*Observed cases*)

TempMinCasa=23;(*Minimal in-house temperature*)

Sintomatic=1/11;(*Symptomatic cases*)

CortePluvio=1;(*Rainfall cutoff*)

(*Introduction of meteorological data*)

L=WeatherData["Campeche", "MeanTemperature", {{2009, 1}, {2015, 12, 31}, "Week"}];

P=WeatherData["Campeche", "TotalPrecipitation", {{2009, 1, 1}, {2015, 12, 31}, "Week"}];

(*Introducing meteorological data into vectors*)

q=1;

For[n=1,n<Length[L],n++,

If[IntegerQ[n/52] False,

Temporal1=Join[Temporal1,{Extract[L,{n,2}]}];

Temporal2=Join[Temporal2,{Extract[P,{n,2}]}];

,

Temperatura=Join[Temperatura,{Max[Temporal1]}];

Lluvias=Join[Lluvias,{Max[Temporal2]}];

Temporal1={};

Temporal2={};

q=q+1;

]

];

PluProm=Mean[Lluvias];

TempMax=Mean[Temperatura];

For[n=1,n<loops,n++,

(*Transforming weekly data to daily data, average of weekly meteorological data*)

Temp=Extract[L,{Round[n/7]+1,2}];

Preci=Extract[P,{Round[n/7]+1,2}];

(*Defining in-house temperature*)

If[Temp<TempMinCasa,TempIn=TempMinCasa,TempIn=Temp-(1.3^(Temp-TempMinCasa)/2^-1)];

(*variable polynomial deffinition*)

Surb=-90.664+9.54TempIn-0.1855TempIn^2^;

EggsNumb=-71.066+7.597 Temp-0.1428 Temp^2^;

TiempoHuevo=37.066-2.0853Temp+0.0319 Temp^2^;

TiempoLarva=55.499-2.8617 Temp+0.0411 Temp^2^;

TiempoPupa=18.786-1.006 Temp+0.0148 Temp^2^;

τ=Round[6000*Sqrt[0.3/(2*Pi)]Exp[-0.3(TempIn-5.9)^2^/TempIn]];

(*Control to cutoff accumulated dengue cases by year*)

If[IntegerQ[n/365] True,Registro=Registro*(1-DismCob),Registro=Registro];

CorrObs=Sintomatic*Registro*TamanoPob/H;

(*Variable definitions*)

k_1_=k_12_*EggsNumb/2;

k_2_=1/TiempoHuevo;

k_3_=0.38/TiempoHuevo;

k_4_=1/TiempoLarva;

k_5_= λ 0.25/TiempoLarva;

k_6_=0.05;

k_7_=1/TiempoPupa;

k_8_=0.09k_7_;

k_9_=0.67*4/7*0.8;

k_10_=1/Surb;

k_11_=1.56*k_10_;

k_12_=0.33*2.1*0.6*(1/k_11_-τ)*UnitStep[1/k_11_-τ]*k_11_*0.5;

k_13_=0.99*k_17_;

k_14_=1/7;

k_15_=0.00000065;

k_16_=1/(6*365);

k_17_=(1-k_19_);

k_18_=UnitStep[Preci-CortePluvio];

k_19_=0.018;

τ=3;

l=(H-(x_9_[t]+x_10_[t]))/H;

s=1-l;

λ=1-(0.1389-0.0136*Preci);

m=0;

(*Equations*)

X1=x_1_'[t] k_1_ (x_7_[t]+(1-m) x_8_[t])+k_18_x_11_[t]-(k_2_+k_17_+k_3_) x_1_[t]; (*Healthy eggs*)

X2=x_2_'[t] m k_1_ x_8_[t]+k_18_ x_12_[t]-(k_2_+k_18_+k_3_) x_2_[t]; (*Infected eggs*)

X3=x_3_'[t] k_2_ x_1_[t]-(k_4_+k_5_+k_6_ x_3_[t]) x_3_[t]; (*Healthy larva*)

X4=x_4_'[t] k_2_ x_2_[t]-(k_4_+k_5_+k_6_ x_4_[t]) x_4_[t]; (*Infected larva*)

X5=x_5_'[t] k_4_ x_3_[t]-(k_7_+k_8_) x_5_[t]; (*Healthy pupa*)

X6=x_6_'[t] k_4_ x_4_[t]-(k_7_+k_8_) x_6_[t]; (*Infected pupa*)

X7=x_7_'[t] k_7_ x_5_[t]-(k_9_ x_9_[t]+k_10_)x_7_[t]; (*Healthy mosquito*)

X8=x_8_'[t] k_7_ x_6_[t- λ]+k_9_ x_9_[t- λ] x_7_[t- λ]-k_11_ x_8_[t]; (*Infected mosquito*)

X9=x_9_'[t] k_12_ (l(H-(x_9_[t]+x_10_[t])))/(s x_9_[t]+l(H-(x_9_[t]+x_10_[t]))) x_8_[t]-(k_13_+k_14_) x_9_[t]; (*Infected human*)

X10=x_10_'[t] k_14_ x_9_[t]-(k_16_+k_15_) x_10_[t]; (*Immune human*)

X11=x_11_'[t] k_17_ x_1_[t]-(k_18_+ k_19_)x_11_[t]; (*Resting healthy eggs*)

X12=x_12_'[t] k_17_ x_2_[t]-(k_18_+k_19_)x_12_[t]; (*Resting infected eggs*)

(*Solving equations*)

sol=NDSolve[{X1,X2,X3,X4,X5,X6,X7,X8,X9,X10,X11,X12,x_1_[0] 0,x_2_[0] 0,x_3_[0] 0,x_4_[0] 0,x_5_[0] 0,x_6_[0] 0,x_7_[0] 0.99,x_8_[0] 0.01,x_9_[0] 0,x_10_[0] 0,x_11_[0] 0,x_12_[0] 0},{x_1_,x_2_,x_3_,x_4_,x_5_,x_6_,x_7_,x_8_,x_9_,x_10_,x_11_,x_12_},{t,n,n+1}];

(*Collecting outcomes*)

If[n<AnosEstabilizar*365,Continue,

Sol1=Join[Sol1,{Evaluate[x_9_[n]/.sol][[1]]}];

Sol2=Join[Sol2,{Temp}];

Sol3=Join[Sol3,{Preci}];

Sol4=Join[Sol4,{TempIn}];

Tempera=Join[Tempera,{Temp}];

Pluvio=Join[Pluvio,{Preci}];

]

];

(*Control to cutoff accumulated dengue cases by year*)

p1=Sum[Sol1[[i]]*CorrObs,{i,1,365}];

p2=Sum[Sol1[[i]]*CorrObs,{i,366,2*365}];

p3=Sum[Sol1[[i]]*CorrObs,{i,2*365+1,3*365}];

(*Projecting the incidence*)

Sol5=Join[Sol5,{Sol1[[1]]*CorrObs/(TamanoPob/100000)}];

(*Projecting the prevalence*)

For[i=2,i<AnosSim*365,i++,

Sol5=Join[Sol5,If[IntegerQ[i/365] True,{0},{Sol5[[i-1]]+Sol1[[i]]*CorrObs/(TamanoPob/100000)}]];

];

(*Graphics*)

B1=ListLinePlot[{Sol2,Sol4},PlotStyle→{Directive[Black],Directive[Red]},Frame→True,FrameTicksStyle→Directive[16],PlotRange→{12,32},FrameLabel→{None,Style["Temperature",FontSize→18]},Axes→False,FrameTicks→{{Automatic,None},{{0,{1 365,"2013"},{2 365,"2014"},{3 365,"2015"}},None}}];

B2=ListLinePlot[Sol3,PlotStyle→{Directive[Black]},Frame→True,FrameTicksStyle→Directive[16],PlotRange→{0,18},FrameLabel→{None,Style["Precipitation",FontSize→18]},Axes→False,FrameTicksStyle→Directive[16],FrameTicks→{{Automatic,None},{{0,{1 365,"2013"},{2 365,"2014"},{3 365,"2015"}},None}}];

B3=ListPlot[Sol5,Filling->Axis,FillingStyle→Blue,PlotStyle→{Directive[Black]},Frame→True,PlotStyle→PointSize[1],FrameLabel→{Style["Time (years)",FontSize→18],Style["Incidence rate",FontSize→18]},Axes→False,FrameTicksStyle→Directive[16],FrameTicks→{{Automatic,None},{{0,{1 365,"2013"},{2 365,"2014"},{3 365,"2015"}},None}}];

B4=BarChart[{Labeled[{p1,TR1},Style["2013",FontSize→16]],Labeled[{p2,TR2},Style["2014",FontSize→16]],Labeled[{p3,TR3},Style["2015",FontSize→16]]},FrameTicks→{{Automatic,None},{None,None}},Frame→True,ChartStyle→{Blue,Black},FrameTicksStyle→Directive[16],FrameLabel→{Style["\n"<>"Time (years)",FontSize→18],Style["Number of cases",FontSize→18]}];

GraphicsGrid[{{B1,B2},{B3,B4}},Spacings→{0,0}]

References

1. Panigrahi KS, Barik TK, Mohanty S, Tripathy NK (2014) Laboratory Evaluation of Oviposition Behavior of Field Collected Aedes Mosquitoes. Journal of Insects: 8.

2. Harrington LC, Fleisher A, Ruiz-Moreno D, Vermeylen F, Wa CV, et al. (2014) Heterogeneous feeding patterns of the dengue vector, Aedes aegypti, on individual human hosts in rural Thailand. PLoS Negl Trop Dis 8: e3048.

3. Guzman MG, Halstead SB, Artsob H, Buchy P, Farrar J, Gubler, D. J., et al. (2010) Dengue: a continuing global threat. Nat Rev Microbiol 8: S7-16.

4. Vaughn DW, Green S, Kalayanarooj S, Innis BL, Nimmannitya S, Suntayakorn, S., et al. (2000) Dengue viremia titer, antibody response pattern, and virus serotype correlate with disease severity. J Infect Dis 181: 2-9.

5. Endy TP, Nisalak A, Chunsuttitwat S, Vaughn DW, Green S, Ennis, F. A., et al. (2004) Relationship of preexisting dengue virus (DV) neutralizing antibody levels to viremia and severity of disease in a prospective cohort study of DV infection in Thailand. J Infect Dis 189: 990-1000.

6. Marinho RA, Beserra EB, Bezerra-Gusmao MA, Porto Vde S, Olinda RA, Dos Santos, C. A. (2016) Effects of temperature on the life cycle, expansion, and dispersion of Aedes aegypti (Diptera: Culicidae) in three cities in Paraiba, Brazil. J Vector Ecol 41: 1-10.

7. Maciel-de-Freitas R, Koella JC, Lourenco-de-Oliveira R (2011) Lower survival rate, longevity and fecundity of Aedes aegypti (Diptera: Culicidae) females orally challenged with dengue virus serotype 2. Trans R Soc Trop Med Hyg 105: 452-458.

8. Chadee DD (2013) Resting behaviour of Aedes aegypti in Trinidad: with evidence for the re-introduction of indoor residual spraying (IRS) for dengue control. Parasit Vectors 6: 255.

9. Zhang X, Tang S, Cheke RA, Zhu H (2016) Modeling the Effects of Augmentation Strategies on the Control of Dengue Fever With an Impulsive Differential Equation. Bull Math Biol 78: 1968-2010.

10. Horta MA, Bruniera R, Ker F, Catita C, Ferreira AP (2014) Temporal relationship between environmental factors and the occurrence of dengue fever. Int J Environ Health Res 24: 471-481.

11. Chowell G, Diaz-Duenas P, Miller JC, Alcazar-Velazco A, Hyman JM, et al. (2007) Estimation of the reproduction number of dengue fever from spatial epidemic data. Math Biosci 208: 571-589.

12. Chan M, Johansson MA (2012) The incubation periods of Dengue viruses. PLoS One 7: e50972.

13. Esteva L, Vargas C (1999) A model for dengue disease with variable human population. J Math Biol 38: 220-240.

14. Ehelepola ND, Ariyaratne K, Buddhadasa WM, Ratnayake S, Wickramasinghe M (2015) A study of the correlation between dengue and weather in Kandy City, Sri Lanka (2003 -2012) and lessons learned. Infect Dis Poverty 4: 42.

15. Hancock PA, White VL, Callahan AG, Godfray CHJ, Hoffmann AA, Ritchie SA (2016) Density-dependent population dynamics in Aedes aegypti slow the spread of wMel Wolbachia. J Appl Ecol: 785-793.

16. Bousema T, Dinglasan RR, Morlais I, Gouagna LC, van Warmerdam T, Awono-Ambene PH et al. (2012) Mosquito feeding assays to determine the infectiousness of naturally infected Plasmodium falciparum gametocyte carriers. PLoS One 7: e42821.

17. Duong V, Lambrechts L, Paul RE, Ly S, Lay RS, Long, K. C., et al. (2015) Asymptomatic humans transmit dengue virus to mosquitoes. Proc Natl Acad Sci U S A 112: 14688-14693.

18. Carrington LB, Simmons CP (2014) Human to mosquito transmission of dengue viruses. Front Immunol 5: 290.

19. Vazeille M, Rosen L, Mousson L, Failloux AB (2003) Low oral receptivity for dengue type 2 viruses of Aedes albopictus from Southeast Asia compared with that of Aedes aegypti. Am J Trop Med Hyg 68: 203-208.
